# Plant-Based Diets and Risk of Hip Fracture in Postmenopausal Women

**DOI:** 10.1001/jamanetworkopen.2024.1107

**Published:** 2024-02-29

**Authors:** Mercedes Sotos-Prieto, Fernando Rodriguez-Artalejo, Teresa T. Fung, Haakon E. Meyer, Frank B. Hu, Walter C. Willett, Shilpa N. Bhupathiraju

**Affiliations:** 1 Department of Preventive Medicine and Public Health, School of Medicine, Universidad Autónoma de Madrid, Madrid, Spain; 2 CIBERESP (CIBER of Epidemiology and Public Health), Madrid, Spain; 3 IMDEA-Food Institute, Campus of International Excellence and University of Madrid and Spanish National Research Council (CEI UAM-CSIC), Madrid, Spain; 4 Department of Environmental Health, Harvard T.H. Chan School of Public Health, Boston, Massachusetts; 5 Department of Nutrition, Simmons University, Boston, Massachusetts; 6 Channing Division of Network Medicine, Department of Medicine, Brigham & Women’s Hospital and Harvard Medical School, Boston, Massachusetts; 7 Department of Community Medicine and Global Health, University of Oslo, Oslo, Norway; 8 Division of Mental and Physical Health, Norwegian Institute of Public Health, Oslo, Norway; 9 Department of Nutrition, Harvard T.H. Chan School of Public Health, Boston, Massachusetts

## Abstract

**Importance:**

Previous research has found that vegetarian diets are associated with lower bone mineral density and higher risk of fractures, but these studies did not differentiate the quality of the plant-based foods.

**Objective:**

To examine the association between the quality of plant-based diets (not necessarily vegan but also omnivorous) and hip fracture risk among postmenopausal women in the Nurses’ Health Study.

**Design, Setting, and Participants:**

This cohort study analyzed data from 70 285 postmenopausal women who participated in the US Nurses’ Health Study from 1984 through 2014. Data were analyzed from January 1 to July 31, 2023.

**Main outcomes and Measures:**

Hip fractures were self-reported on biennial questionnaires. Diet was assessed every 4 years using a validated semiquantitative food frequency questionnaire. Plant-based diet quality was assessed using 2 previously established indices: the healthful Plant-Based Diet Index (hPDI), for which healthy plant foods (whole grains, fruits, vegetables, nuts, legumes, vegetable oils, and tea or coffee) received positive scores, whereas less healthy plant foods (fruit juices, sweetened beverages, refined grains, potatoes, and sweets or desserts) and animal foods received reversed scores; and the unhealthful Plant-Based Diet Index (uPDI), for which positive scores were given to less healthy plant foods and reversed scores to healthy plant and animal foods. Quintile scores of 18 food groups were summed, with a theoretical range for both indices of 18 to 90 (highest adherence). Cox proportional hazards regression with time-varying covariates was used to compute hazard ratios (HRs) and 95% CIs for hip fracture.

**Results:**

In total, 70 285 participants (mean [SD] age, 54.92 [4.48] years; 100% White women) were included, and 2038 cases of hip fracture were ascertained during the study and for up to 30 years of follow-up. Neither the hPDI (HR for highest vs lowest quintile, 0.97 [95% CI, 0.83-1.14]) nor the uPDI (HR for highest vs lowest quintile, 1.02 [95% CI, 0.87-1.20]) for long-term diet adherence was associated with hip fracture risk. However, when examining recent intake for the highest vs lowest quintiles, the hPDI was associated with 21% lower risk of hip fracture (HR, 0.79 [95% CI, 0.68-0.92]; *P* = .02 for trend), and the uPDI was associated with 28% higher risk (1.28 [95% CI, 1.09-1.51]; *P* = .008 for trend).

**Conclusions and Relevance:**

Findings of this cohort study indicated that long-term adherence to healthful or unhealthful plant-based diets as assessed by hPDI and uPDI scores was not associated with hip fracture risk. Future research should clarify whether the associations observed with recent dietary intake are due to short-term effects of these dietary patterns, reverse causality, or both.

## Key Points

**Question:**

Are plant-based diets associated with risk of hip fractures in postmenopausal women?

**Findings:**

In this cohort study that included 70 285 postmenopausal women in the US, long-term adherence to a plant-based diet was not associated with the risk of hip fracture. However, comparing lowest to highest quintiles of Plant-Based Diet Index scores, the most recent intake of a healthy plant-based diet was associated with 21% lower risk of fracture, whereas the most recent intake of an unhealthy plant-based diet was associated with 28% higher risk of fracture.

**Meaning:**

Findings of this cohort study suggest that following a plant-based diet over time appears safe regarding the risk of hip fracture.

## Teaser

This cohort study evaluates data from the Nurses’ Health Study to assess whether there is an association between the quality of plant-based diets and hip fracture risk among postmenopausal women.

## Introduction

Bone fractures are a public health issue^1^ among older adults because bone mass decreases with age, especially in postmenopausal women. Worldwide, 1 in 3 women older than 50 years will experience a fragility fracture,^2^ which is a leading cause of long-term morbidity.^3^

Adequate nutrient intake plays an essential role in bone mineralization. Some nutrients, such as calcium, vitamin D, or vitamin K, have shown an association with reduced bone loss.^4,5^ Although a systematic review of clinical trials and observational studies showed that dietary calcium intake or dairy consumption was not associated with risk of fractures,^5,6,7,8,9,10^ a recent study found that high dietary calcium intake in the context of a Mediterranean-style diet was associated with lower risk of fractures.^11^ Other nutrients, such as proteins, vitamin B_12_, zinc, or fatty acids, have also been associated with bone health in some investigations but not all.^12,13,14^ In fact, the combined, very high intake of vitamins B_6_ and B_12_ has been associated with increased risk of hip fractures in postmenopausal women.^15^

Plant-based diets, characterized by higher consumption of plant foods and lower or no intake of animal foods, have raised concerns about their potential harmful effect on bone health related to the shortfalls of a vegetarian diet.^16^ A systematic review found that vegetarians, but particularly vegans (defined as those with no consumption of any source of animal food), had higher risk of fractures and lower bone mineral density compared with omnivores.^14^ Likewise, a previous study in the European Prospective Investigation Into Cancer and Nutrition (EPIC)–Oxford cohort found that compared with meat eaters, fish eaters and vegetarians had higher risk of hip fractures.^17^ However, none of those studies assessed the quality of the plant-based diets, which is important because not all plant-based diets are healthy.^16^ Previous research supports the benefits of a healthful plant-based diet for different health outcomes.^18,19,20,21,22^ However, evidence is limited about the association between the Plant-Based Diet Index (PDI) and risk of fractures. Therefore, our aim was to study the association between plant-based diet quality and risk of hip fracture among postmenopausal women in the Nurses’ Health Study (NHS). Of note is that the PDI does not necessarily correspond to a vegan or vegetarian diet. Instead, the PDI is a composite score assessing the quality of plant-derived foods, and it does not exclude the consumption of animal foods but rather scores them reversed.

## Methods

### Study Population

The NHS is a prospective cohort study that started in 1976 and included 121 700 female registered nurses in the United States aged 30 to 55 years.^23^ Every 2 years, participants provided information about medical history and health-related information. In addition, every 4 years, a food frequency questionnaire (FFQ) was sent to obtain dietary information. Follow-up rates exceed 90%. For the present analysis, only White postmenopausal women were included at baseline (1984). Otherwise, women entered the questionnaire cycle when they reached menopause, including surgical menopause. Women with different races and ethnicities were excluded because their numbers were very low. We excluded participants at entry with cancer (as the medical treatment may affect bone mineral density), with a previously reported hip fracture or osteoporosis (because participants at higher risk of fracture may change their diet), or with missing diet information (eFigure in Supplement 1). The NHS protocol was approved by the institutional review boards of the Brigham and Women’s Hospital and Harvard T.H. Chan School of Public Health. Completion and return of the self-administered questionnaires constituted informed consent. This report follows the Strengthening the Reporting of Observational Studies in Epidemiology (STROBE) reporting guideline for observational studies.

### Assessment of PDIs

Dietary data were collected with a self-administered semiquantitative FFQ^24^ every 4 years between 1984 and 2010. Participants were asked how often on average during the previous 12 months they had consumed each food of a standard portion size. Reliability and validity of the FFQ have been reported elsewhere.^25^

Two types of a PDIs were computed to consider the quality of the foods included, a healthful PDI (hPDI) and an unhealthful PDI (uPDI). Details about food groups and scoring criteria for both indices have been previously reported.^26,27^ In brief, all food items were classified into 18 food groups based on nutrients and culinary similarities, and these groups were classified into 3 larger categories of healthy plant foods, less healthy plant foods, and animal foods. The 18 food groups were ranked into quintiles, and each quintile was given a positive or reversed score, with a range from 1 to 5 (eTable 1 in Supplement 1). For the hPDI, the healthy plant food groups were given positive scores, whereas the less healthy plant food and animal food groups were given a reversed score. For the uPDI, positive scores were given to less healthy plant food groups, and reversed scores to healthy plant food groups and animal food groups. The quintile scores of the 18 food groups were summed, so the theoretical range for both the hPDI and the uPDI was from 18 to 90 (highest adherence).

### Assessment of Hip Fractures

Participants in the study reported any hip fractures they experienced, including the month and year that they occurred, on biennial questionnaires. Participants also provided details about the circumstances surrounding the fracture, which were used to determine the level of trauma. Fractures caused by high-impact trauma, such as motor vehicle crashes, horseback riding, skiing, and similar activities, were not included. A validation study using medical records conducted within the NHS confirmed every reported fracture in all 30 cases examined.^28^ Finally, hip fractures were identified from death records as well.

### Assessment of Other Lifestyle Characteristics

Biannually, we collected information on age, body mass index (BMI; calculated as weight in kilograms divided by height in meters squared), smoking status, and use of thiazide diuretics, furosemide-like diuretics, anti-inflammatory steroids, and postmenopausal hormone treatment. Participants reported use of brand-specific multivitamins; use of supplementation with calcium, vitamin D, and retinol; and receipt of a diagnosis of diabetes. Leisure-time physical activity was assessed with up to 10 activities^29^ from which metabolic equivalent of task hours per week were calculated.

### Statistical Analysis

Participants were followed up from the date of the questionnaire cycle that they first reported being in menopause (FFQ in 1984 as the first diet assessment) until the date of first hip fracture, death from hip fracture, last questionnaire response, or the end of follow-up in 2014, whichever came first. For the primary analysis, to capture the long-term diet, we used a cumulative mean of the hPDI and uPDI scores (ie, the mean diet across points of data collection). In a secondary analysis, to assess the recent diet, we used dietary information from the FFQ prior to the hip fracture or the end of follow-up, whichever was earlier. To assess the baseline diet, we used dietary information from the FFQ when the participant first reported being postmenopausal.

We used Cox proportional hazards regression models (with time-varying covariates when the exposure was the long-term dietary indices using the updated information for covariates in each cycle [ie, BMI, physical activity, energy intake, and alcohol consumption] and baseline covariates when the exposure was the baseline diet) to compute the hazard ratios (HRs) and 95% CIs of hip fracture according to quintiles of the hPDI and uPDI, with the lowest quintile being the reference group. Linear trends were evaluated using quintiles of the indices as a continuous variable. Additionally, we estimated the risk of hip fracture per 10-unit increase in the hPDI and uPDI. We sequentially adjusted for covariates to assess their association with the HR. In model 1, we adjusted for age and energy intake. Model 2 additionally adjusted for BMI and height (in centimeters). In model 3, we further adjusted for physical activity, smoking, alcohol intake, and history of diabetes. In our final model (model 4), we adjusted for medication and supplement use that can affect bone mineral density, including the use of thiazides, furosemide-like diuretics, oral anti-inflammatory steroids, and multivitamins, postmenopausal hormone treatment, and supplemental intakes of calcium, retinol, and vitamin D.

In secondary and sensitivity analyses, we first assessed the associations between individually modified PDIs and risk of hip fracture, including an hPDI with a positive score for yogurt, fish, and poultry. We then estimated the association of specific healthy and less healthy plant foods and animal foods with the risk of hip fractures. We next evaluated diet indices reported at different latencies (2, 6, and 10 years) before a hip fracture occurrence to account for potential bias resulting from changes in diet because of signs of bone demineralization or osteoporosis. We also conducted stratified analyses to examine whether any association between the PDI and hip fracture differed by age, BMI, or physical activity; the likelihood ratio test comparing regression models with vs without an interaction term was used to assess the interaction. We addressed residual confounding using the E-value for the null hypothesis^30^

Analyses were performed using SAS software for UNIX, version 9.4 (SAS Institute Inc). A 2-sided *P* < .05 was considered statistically significant. Data were analyzed from January 1 to July 31, 2023.

## Results

Of 70 285 participants included in the present study, we ascertained 2038 cases of hip fractures in the NHS during follow-up up to 30 years. The age-adjusted baseline characteristics of the participants according to quintiles of the hPDI and uPDI are presented in Table 1. Overall, at baseline, the mean (SD) age of participants was 54.92 (4.48) years, and 100% were White women. The mean (SD) hPDI was 54.33 (7.33) points, and it was 54.38 (7.70) points for the uPDI. The mean (SD) energy consumption was 1743.65 (521.07) kcal, and the mean (SD) BMI was 25.69 (4.85). Compared with participants with lower hPDI scores, individuals with higher hPDI scores were leaner, more physically active, less likely to be smokers, more likely to use vitamin and calcium supplements, had higher intakes of dietary calcium and healthy plant foods, and had lower intake of less healthy plant foods. Conversely, those with higher scores in the uPDI were less physically active, more likely to smoke, had lower calcium and alcohol intake, were less likely to use vitamin supplements, had higher consumption of less healthy plant foods, and had lower consumption of healthy plant foods. Compared with individuals with the least adherence to the hPDI and uPDI, those with the highest adherence to the hPDI and uPDI had lower energy intake and lower vitamin D supplemental intake.

**Table 1.** Age-Adjusted Baseline Characteristics According to Quintiles of Plant-Based Diet Indexes in the Nurses’ Health Study

Characteristic	Plant-based diet index, quintile^a^			
	Healthful		Unhealthful	
	1 (Lowest adherence)	5 (Highest adherence)	1 (Lowest adherence)	5 (Highest adherence)
hPDI, mean (SD), score	43.7 (3.1)	64.6 (3.3)	58.2 (6.7)	50.7 (6.5)
uPDI, mean (SD), score	58.3 (6.9)	50.4 (7.0)	43.4 (3.3)	65.6 (3.3)
Age at entry to study, mean (SD), y	54.2 (4.6)	55.7 (4.2)	55.3 (4.3)	54.6 (4.7)
BMI, mean (SD)	26.4 (5.4)	25.1 (4.3)	26.0 (4.8)	25.4 (4.9)
Height, mean (SD), cm	164.1 (6.0)	163.6 (6.1)	164.2 (6.1)	163.4 (6.1)
History of diabetes, No. (%)	464 (7.99)	531 (7.03)	554 (8.52)	456 (6.76)
Physical activity, mean (SD), MET h/wk	11.8 (13.6)	17.8 (18.0)	17.8 (17.5)	11.2 (14.0)
Current smoker, No. (%)	1264 (21.8)	1297 (17.1)	1067 (16.4)	1577 (23.4)
Alcohol intake, mean (SD), g/d	6.3 (10.7)	6.3 (10.7)	7.4 (11.1)	5.1 (10.2)
Energy intake, mean (SD), kcal/d	2085 (495)	1475 (438)	1986 (503)	1514.7 (472.6)
Total calcium, mean (SD), mg^b^	800.0 (386.7)	1129.4 (576.4)	1120.2 (469.9)	868.9 (495.6)
Mean (SD) servings/d				
Healthy plant foods	7.69 (2.97)	11.13 (3.92)	12.48 (3.77)	6.43 (2.52)
Whole grains	0.76 (0.86)	1.46 (1.28)	1.63 (1.26)	0.63 (0.79)
Fruits^c^	1.01 (0.86)	1.86 (1.23)	2.05 (1.20)	0.91 (0.75)
Vegetables	2.51 (1.27)	3.52 (1.89)	4.11 (1.87)	1.94 (1.03)
Nuts	0.18 (0.26)	0.25 (0.37)	0.30 (0.39)	0.13 (0.25)
Legumes	0.16 (0.15)	0.25 (0.25)	0.29 (0.25)	0.13 (0.14)
Less healthy plant foods	6.40 (2.69)	2.91 (1.80)	3.84 (2.26)	5.14 (2.66)
Refined grains	2.42 (1.54)	1.16 (1.07)	1.54 (1.29)	1.91 (1.42)
Potatoes	0.69 (0.41)	0.33 (0.29)	0.43 (0.33)	0.56 (0.40)
Sugar-sweetened beverages	0.56 (0.76)	0.09 (0.30)	0.13 (0.33)	0.47 (0.73)
Sweets and desserts	1.82 (1.45)	0.79 (0.89)	1.05 (1.09)	1.46 (1.36)
Fruit juice	0.91 (0.78)	0.55 (0.68)	0.69 (0.74)	0.75 (0.74)
Animal foods	6.27 (2.21)	4.13 (1.76)	6.25 (2.14)	3.98 (1.75)
Dairy	2.31 (1.42)	1.73 (1.22)	2.51 (1.41)	1.51 (1.16)
Eggs	0.43 (0.33)	0.27 (0.29)	0.45 (0.34)	0.25 (0.27)
Fish	0.30 (0.23)	0.33 (0.30)	0.47 (0.33)	0.19 (0.16)
Meat	1.98 (0.80)	1.32 (0.70)	1.84 (0.83)	1.42 (0.72)
Red meat	1.49 (0.74)	0.78 (0.56)	1.15 (0.71)	1.07 (0.67)
Poultry	0.48 (0.36)	0.54 (0.44)	0.69 (0.51)	0.35 (0.28)
Thiazide, No. (%)	13.0 (754)	13.3 (1008)	15.0 (976)	12.2 (822)
Use of furosemide-like diuretics, No. (%)	91 (1.57)	79 (1.04)	83 (1.28)	103 (1.53)
Anti-inflammatory steroids, No. (%)	99 (1.71)	96 (1.27)	85 (1.30)	109 (1.61)
Multivitamin supplement, No. (%)	1972 (35.72)	3788 (43.96)	2927 (44.98)	2293 (34.02)
Menopausal hormone use, No. (%)	1995 (34.4)	4398 (33.5)	2310 (35.5)	2130 (31.6)
Supplemental calcium, mg/d	189 (3349)	329.3 (437)	308 (422)	206 (361)
Supplemental vitamin D, IU/d	299 (200)	170.1 (244)	173 (246)	118 (201)
Supplemental retinol, IU/d	1758 (3695)	2879.5 (5210)	2864 (5329)	1755 (3614)

Abbreviations: BMI, body mass index (calculated as weight in kilograms divided by height in meters squared); hPDI, healthful plant-based diet; MET, metabolic equivalent of task; uPDI, unhealthful plant-based diet.

^a^ Quintile scores of 18 food groups were summed; thus, the theoretical range for the hPDI and for the uPDI was 18 to 90 (highest adherence).

^b^ Total calcium intake included supplemental sources.

^c^ Excluding fruit juices.

Long-term hPDI or uPDI scores were not associated with hip fracture risk; the adjusted HR for the highest quintile (Q5) vs the lowest quintile (Q1) was 0.97 (95% CI, 0.83-1.14) for the hPDI and 1.02 (95% CI, 0.87-1.20) for the uPDI (Table 2). However, when examining recent intake, we found that the hPDI was associated with 21% lower risk of hip fracture (HR, 0.79 [95% CI, 0.68-0.92]; *P* = .02 for trend), whereas the uPDI was associated with 28% higher risk (HR, 1.28 [95% CI, 1.09-1.51]; *P* = .008 for trend) (Table 3). Similarly, using only diet at baseline (first diet assessment when a woman reached menopause), as other studies have used,^31,32^ we found a higher risk of hip fracture with higher scores in the uPDI, although the test for trend was not statistically significant (HR for quintile 5, 1.20 [95% CI, 1.03-1.40]; *P* = .06 for trend). When adjusted for physical activity and BMI, the associations were slightly attenuated.

**Table 2.** Risks of Hip Fracture According to Quintiles of Cumulative Plant-Based Diet Index Scores in Postmenopausal Women in the Nurses’ Health Study

Diet	Relative risk (95% CI)					*P* value for trend
	Quintile 1 (lowest)	Quintile 2	Quintile 3	Quintile 4	Quintile 5	
**Healthful plant-based diet**						
No. of cases/person-years	339/304 430	446/313 816	396/312 667	445/308 613	412/298 575	NA
Model 1^a^	1 [Reference]	1.09 (0.95-1.26)	0.93 (0.80-1.08)	1.01 (0.87-1.17)	0.93 (0.79-1.08)	.15
Model 2^b^	1 [Reference]	1.08(0.97-1.24)	0.91(0.78-1.06)	0.97 (0.83-1.12)	0.86 (0.74-1.01)	.02
Model 3^c^	1 [Reference]	1.11(0.96-1.29)	0.96 (0.83-1.12)	1.04 (0.90- 1.21)	0.96 (0.82-1.13	.38
Model 4^d^	1 [Reference]	1.13 (0.97-1.30)	0.98 (0.84-1.13)	1.06 (0.91-1.23)	0.97 (0.83-1.14)	.45
**Unhealthful plant-based diet**						
No. of cases/person-years	350/317 244	383/317 206	420/311 927	461/306 807	424/284 917	NA
Model 1^a^	1 [Reference]	1.02 (0.88-1.18)	1.08 (0.93-1.25)	1.18 (1.02-1.36)	1.16 (0.99-1.34)	.01
Model 2^b^	1 [Reference]	1.01 (0.87-1.17)	1.06 (0.92-1.22)	1.14 (0.99-1.32)	1.12 (0.96-1.30)	.04
Model 3^c^	1 [Reference]	0.99 (0.86-1.15)	1.03 (0.89-1.19)	1.09 (0.93-1.26)	1.04 (0.88-1.21)	.38
Model 4^d^	1 [Reference]	1.00 (0.86-1.16)	1.03 (0.89-1.19)	1.08 (0.93-1.26	1.02 (0.87-1.20	.48

Abbreviation: NA, not applicable.

^a^ Model 1 includes age and energy intake.

^b^ Model 2 includes model 1 plus body mass index and height.

^c^ Model 3 includes model 2 plus physical activity, smoking, alcohol, and history of diabetes.

^d^ Model 4 includes model 3 plus (1) use of postmenopausal hormones, thiazides, furosemide-like diuretics, anti-inflammatory steroids, and multivitamin supplements and (2) supplemental intakes of calcium, vitamin D, and retinol.

**Table 3.** Risk of Hip Fracture According to Quintiles of Most Recent and Baseline Plant-Based Diet Index Scores in the Nurses’ Health Study

Condition	Relative risk (95% CI)					*P* value for trend
	Quintile 1 (lowest)	Quintile 2	Quintile 3	Quintile 4	Quintile 5	
**Most recent**						
Healthful plant-based diet						
No. of cases/persons-years	395/289 178	383/295 418	369/298 594	413/284 796	328/282 955	NA
Model 1^a^	1 [Reference]	0.93 (0.81-1.07)	0.88 (0.76-1.02)	0.98 (0.85-1.13)	0.80 (0.69-0.93)	.03
Model 2^b^	1 [Reference]	0.92 (0.79-1.06)	0.86 (0.74-0.99)	0.94 (0.82-1.09)	0.75 (0.64-0.88)	.002
Model 3^c^	1 [Reference]	0.94 (0.81-1.08)	0.88 (0.76-1.02)	0.98 (0.84-1.13)	0.79 (0.68-0.93)	.02
Model 4^d^	1 [Reference]	0.94 (0.81-1.08)	0.88 (0.76-1.02)	0.98 (0.85-1.13)	0.79 (0.68-0.92)	.02
Unhealthful plant-based diet						
No. of cases/persons-years	258/293 476	367/302 505	362/286 129	433/285 710	468/283 120	NA
Model 1^a^	1 [Reference]	1.24 (1.05-1.45)	1.22 (1.04-1.44)	1.34 (1.14-1,57)	1.36 (1.15-1.59)	<.001
Model 2^b^	1 [Reference]	1.23 (1.04-1.44)	1.21 (1.03-1.43)	1.33 (1.14-1.56)	1.34 (1.14-1.57)	<.001
Model 3^c^	1 [Reference]	1.23 (1.05-1.45)	1.20 (1.02-1.41)	1.30 (1.10-1.52)	1.29 (1.09-1.52)	.006
Model 4^d^	1 [Reference]	1.23 (1.04-1.44)	1.20 (1.01-1.41)	1.29 (1.10-1.52)	1.28 (1.09-1.51)	.008
**Baseline**						
Healthful plant-based diet						
No. of cases/persons-years	328/276 400	452/345 968	462/325 548	344/278 482	452/311 703	NA
Model 1^a^	1 [Reference]	1.02 (0.89-1.18)	1.06 (0.91-1.23)	0.87 (0.74-1.01)	0.93 (0.80-1.09)	.07
Model 2^b^	1 [Reference]	1.02 (0.88-1.18)	1.04 (0.90-1.21)	0.85 (0.73-1.00)	0.91 (0.78-1.06)	.03
Model 3^c^	1 [Reference]	1.06 (0.92-1.23)	1.11 (0.95- 1.28)	0.92 (0.78-1.07)	1.01 (0.86-1.18)	.40
Model 4^d^	1 [Reference]	1.07 (0.93-1.24)	1.11 (0.96-1.29)	0.92 (0.78-1.08)	1.01 (0.86-1.19)	.43
Unhealthful plant-based diet						
No. of cases/persons-years	370/295 124	447/330 548	369/303 829	437/329 405	415/279 195	NA
Model 1^a^	1 [Reference]	1.11 (0.97-1.28)	1.03 (0.89-1.19)	1.12 (0.97-1.30)	1.27 (1.10- 1.47)	.004
Model 2^b^	1 [Reference]	1.12 (0.97-1.29)	1.04 (0.90-1.20)	1.14 (0.98-1.31)	1.30 (1.12-1.50)	.002
Model 3^c^	1 [Reference]	1.11 (0.96-1.27)	1.01 (0.87-1.17)	1.08 (0.94-1.25)	1.20 (1.04-1.40)	.05
Model 4^d^	1 [Reference]	1.11 (0.96-1.28)	1.01 (0.87-1.17)	1.08 (0.93-1.25)	1.20 (1.03-1.40)	.06

Abbreviation: NA, not applicable.

^a^ Model 1 includes age and energy intake.

^b^ Model 2 includes model 1 plus body mass index and height.

^c^ Model 3 includes model 2 plus physical activity, smoking, alcohol, and history of diabetes.

^d^ Model 4 includes model 3 plus use of postmenopausal hormones, thiazides, furosemide-like diuretics, anti-inflammatory steroids, and multivitamin supplements and supplemental intakes of calcium, vitamin D, and retinol.

In sensitivity analyses, when we assessed long-term adherence to modified healthy plant-based diets, we did not document any associations between hPDI scores and hip fracture risk (eTable 2 in Supplement 1). Likewise, individual servings of food groups in the hPDI and uPDI were not associated with hip fracture risk (eTable 3 in Supplement 1). Latency analyses did not document any associations of hPDI and uPDI with hip fracture risk (eTable 4 in Supplement 1). We found no significant differences between subgroups for interactions by age, BMI, or physical activity (eTable 5 in Supplement 1).

Finally, assuming a reasonable protective risk factor of 0.90, for the hPDI, we obtained an E-value of 1.38, which describes the magnitude of the association an unmeasured confounder would need to have with the hPDI and risk of fracture to move the observed relative risk (RR) from 0.97 to 0.90. Likewise, for the uPDI, for an unmeasured confounder to shift the observed RR estimate of 1.02 to an RR of 1.15 (reasonable high-risk factor), an unmeasured confounder that was associated with both the uPDI and the risk of fracture by an RR of 1.51-fold each could do so, but a weaker confounder could not.

## Discussion

In this cohort study of postmenopausal women in the US, long-term adherence to a plant-based diet was not associated with risk of hip fracture. We, however, found that the most recent hPDI was associated with 21% lower risk of fracture, whereas the most recent uPDI was associated with 28% higher risk of fracture. In addition, higher baseline scores in the uPDI were associated with higher risk of hip fracture.

Studies using repeated dietary measures to examine the associations between long-term usual diet, the nutritionally relevant exposure, and risk of fractures are scarce. An analysis using data from the NHS and the male-counterpart Health Professionals Follow-Up Study showed that of 3 dietary patterns, namely, the Mediterranean diet, Dietary Approaches to Stop Hypertension (DASH), and the Alternate Healthy Eating Index-2010 (AHEI-2010), only the AHEI-2010 was marginally associated with lower risk of hip fracture in women but not in men.^33^ A previous analysis in the same cohorts with repeated measures of diet found no association of a prudent and a Western dietary pattern with risk of fractures.^34^ By contrast, prospective studies using only 1 baseline measurement of diet have shown that a “healthy dietary pattern,”^35,36^ a Mediterranean diet alone^31^ or in combination with calcium supplementation,^11^ or the AHEI-2010^32^ was associated with lower risk of fracture, whereas the Dietary Inflammatory Index^37^ (DII) was associated with higher risk. However, results from the Women’s Health Initiative found that 3-year changes in the DII were not associated with risk of fracture and that only the baseline DII was associated with hip fracture risk among younger White women.^38^ In our study, using only baseline or current diet information, the uPDI was associated with higher risk of hip fracture, and current hPDI was associated with lower risk. These results could be explained by a relatively short-term effect of these dietary patterns potentially influencing the risk of falls (uPDI) due to deficiencies in energy, protein, and micronutrient intake that affect strength and bone health, thus increasing the risk of fracture. But it is also plausible that reverse causation may account for these associations, as individuals with underlying health conditions that predisposed them to higher fracture risk may have changed their diet. In addition, baseline diet may reflect diet early on, which could be an important predictor of bone mineral density when there was more active bone turnover. Further research should confirm these results and clarify their mechanisms.

While most of the healthy dietary patterns share certain components, such as fruits, vegetables, legumes, and cereals, plant-based diets tend to exclude the animal food groups to varying degrees (ie, excluding meat, fish, eggs, dairy, or all of them). Consequently, some researchers argue that plant-based diets or vegetarian diets may fall short of proteins and other nutrients (calcium, vitamin D, and vitamin B_12_) and thus may lead to higher risk of fractures. The role of protein intake in fracture prevention remains controversial. Adequate dietary protein intake is essential for bone and muscle synthesis and repair because bone and muscle are interconnected tissues. A recent umbrella review of systematic reviews found possible evidence for reduced hip fracture risk with high vs low protein intake,^39^ but it remains unclear whether a dose above the current recommendation or type of protein intake (animal or plant protein) may be associated with the results. Animal-based proteins have high amounts of sulfur-containing amino acids, and some research has suggested that these proteins can augment the acid load in the body and lead to bone loss and increased risk of fracture.^40,41^ However, in our study, we found a null association with risk of hip fracture for individual food groups. In addition, scoring positively the consumption of yogurt, fish, or poultry in the hPDI did not change the results. In this regard, the consumption of dairy products and calcium supplementation for the prevention of fractures has been debated in the literature.^42^

The UK Women’s Cohort Study has shown greater risk of hip fracture among vegetarians, but no association was found for pescatarians or occasional meat eaters (<5 servings/week) compared with meat eaters (≥5 servings/week).^43^ Vegans in EPIC showed higher risk of fractures^17,44^; of note, they had lower calcium intake and lower BMI than their nonvegan counterparts, and the fracture risk was higher among people with BMI lower than 22.4. In contrast, in the Adventist Health Study 2, among vegans using supplementation with both vitamin D and calcium, the excess risk of hip fracture disappeared.^45^ Our study differs from the previous ones because our indices do not completely exclude animal consumption but account for the quality of the plant-based foods, and calcium intake was highest among individuals with the greatest adherence to healthy plant-based diets (1129 mg of calcium per day, close to the Recommended Daily Allowance of 1200 mg).

In our study, both physical activity, an established protective factor for hip fractures, and BMI slightly attenuated the association of hPDI and uPDI with hip fracture. Despite previous evidence that lower BMI is associated with higher risk of fracture,^17,46^ our analysis did not show a clear difference between strata. Similarly, the UK Women’s Cohort Study did not find clear evidence of BMI modifying associations between diet groups and hip fracture risk, but vegetarians tended to have lower BMI.^47^ In our study, participants with higher adherence to healthy plant-based diets had lower BMI and higher physical activity.

### Strengths and Limitations

Our study has several strengths, including the large sample size and long follow-up, which allowed for a substantial number of hip fractures, and the use of repeated assessment of diet and lifestyles over time, which enabled calculating diet scores at different time points rather than using only baseline data, as in most previous studies. This study has limitations. Data on diet, lifestyle, and hip fracture were self-reported, but the FFQ has been validated against biomarkers and diet records,^24^ and a validation study with medical records showed that participants accurately reported incidence of hip fracture.^28^ Despite controlling for many potential confounders, residual confounding cannot be ruled out. The use of antiosteoporotic medication was not adjusted for because of lack of information. Finally, our study findings may not be generalizable because we included only postmenopausal White women.

## Conclusions

The results of this cohort study indicated that long-term adherence to a plant-based diet was not associated with hip fracture risk. Future research should clarify whether the results for recent dietary intake are associated with the relatively short-term effects of these dietary patterns, reverse causality, or both.

## Notes

Supplement 1. **eFigure.** Flowchart of participants for the analysis of adherence to plant-based diet indices with risk of fracture **eTable 1.** Food items and criteria for scoring each plant-based diet indices (from the 1984 NHS food frequency questionnaire) **eTable 2.** Relative risks (95% confidence interval) for hip fracture according to quintiles of cumulative modified-healthful plant-based diet scores in the Nurses’ Health Study (n= 70,285 postmenopausal) **eTable 3.** Relative risks (95% confidence interval) for hip fracture according to quintiles of cumulative food categories scores in the Nurses’ Health Study (n= 70,285, postmenopausal) **eTable 4.** Relative risks (95% confidence interval) for hip fracture according to quintiles of cumulative plant-based diet indices with different latency periods in the Nurses’ Health Study **eTable 5.** Relative Risks (95% confidence interval) for hip fracture according to quintiles of cumulative plant-based diet indices in different subgroups in the Nurses’ Health Study

Supplement 2.
Data Sharing Statement
