# Supplementary material for: Plant-Based Diets and Risk of Hip Fracture in Postmenopausal Women
Source: JAMA Netw Open. 2024 Feb 29;7(2):e241107. doi: 10.1001/jamanetworkopen.2024.1107 (PMC10905300; doi:10.1001/jamanetworkopen.2024.1107)
Supplement: Supplement 1. — eFigure. Flowchart of participants for the analysis of adherence to plant-based diet indices with risk of fracture eTable 1. Food items and criteria for scoring each plant-based diet indices (from the 1984 NHS food frequency questionnaire) eTable 2. Relative risks (95% confidence interval) for hip fracture according to quintiles of cumulative modified-healthful plant-based diet scores in the Nurses’ Health Study (n= 70,285 postmenopausal) eTable 3. Relative risks (95% confidence interval) for hip fracture according to quintiles of cumulative food categories scores in the Nurses’ Health Study (n= 70,285, postmenopausal) eTable 4. Relative risks (95% confidence interval) for hip fracture according to quintiles of cumulative plant-based diet indices with different latency periods in the Nurses’ Health Study eTable 5. Relative Risks (95% confidence interval) for hip fracture according to quintiles of cumulative plant-based diet indices in different subgroups in the Nurses’ Health Study [file jamanetwopen-e241107-s001.pdf]

Supplementary Online Content

Sotos-Prieto M, Rodriguez-Artalejo F, Fung TT, et al. Plant-based diets and risk of hip fracture in postmenopausal women. *JAMA Netw Open*. 2024;7(2):e241107. doi:10.1001/jamanetworkopen.2024.1107

eFigure. Flowchart of participants for the analysis of adherence to plant-based diet indices with risk of fracture
eTable 1. Food items and criteria for scoring each plant-based diet indices (from the 1984 NHS food frequency questionnaire)
eTable 2. Relative risks (95% confidence interval) for hip fracture according to quintiles of cumulative modified-healthful plant-based diet scores in the Nurses' Health Study (n= 70,285 postmenopausal)
eTable 3. Relative risks (95% confidence interval) for hip fracture according to quintiles of cumulative food categories scores in the Nurses' Health Study (n= 70,285, postmenopausal)
eTable 4. Relative risks (95% confidence interval) for hip fracture according to quintiles of cumulative plant-based diet indices with different latency periods in the Nurses' Health Study
eTable 5. Relative Risks (95% confidence interval) for hip fracture according to quintiles of cumulative plant-based diet indices with different latency periods in the Nurses' Health Study

This supplementary material has been provided by the authors to give readers additional information about their work

© 2024 Sotos-Prieto M et al. *JAMA Network Open*.

eFigure1. Flowchart of participants for the analysis of adherence to plant-based diet indices with risk of fracture

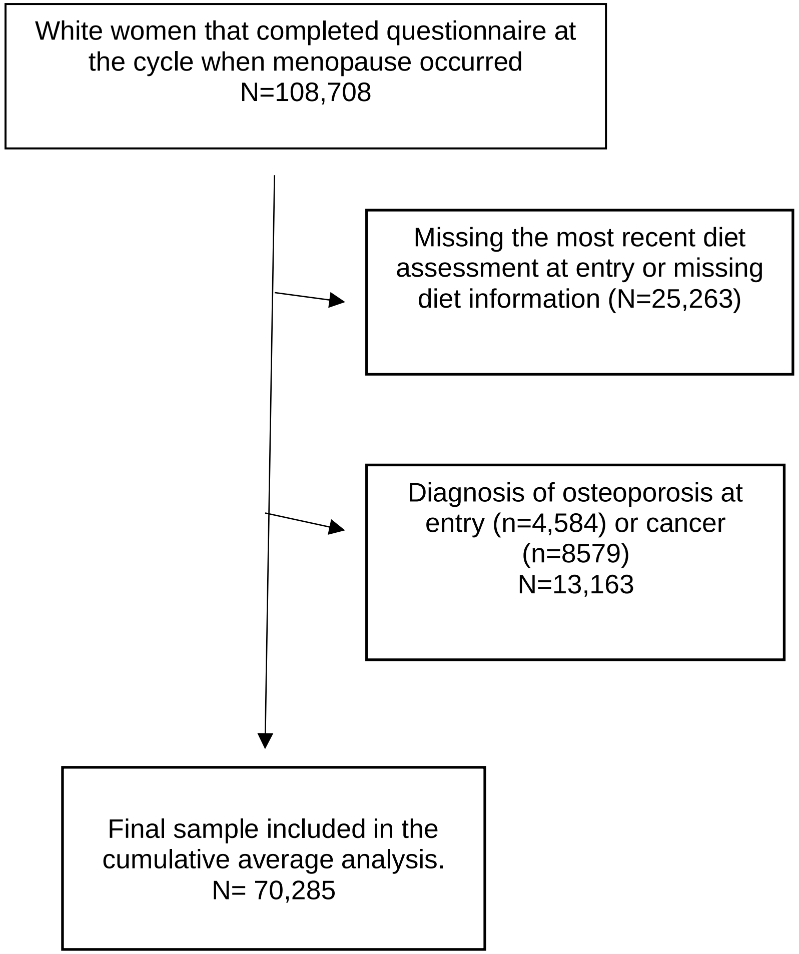

© 2024 Sotos-Prieto M et al. *JAMA Network Open*.

eTable1. Food items and criteria for scoring each plant-based diet indices (from the 1984 NHS food frequency questionnaire)

		hPDI	uPDI
Healthy plant foods			
Whole grains	Whole grain breakfast cereal, other cooked breakfast cereal, cooked oatmeal, dark bread, brown rice, other grains, bran, wheat germ, popcorn	Positive scores	Reverse scores
Fruits	Raisins or grapes, prunes, bananas, cantaloupe, watermelon, avocado, fresh apples, applesauce, fresh pears, canned pears, oranges, grapefruit, strawberries, blueberries, peaches or apricots or plums, canned peaches or apricots, other canned fruit	Positive scores	Reverse scores
Vegetables	Tomatoes, tomato juice, tomato sauce, red chili sauce, broccoli, cauliflower, cooked cabbage, coleslaw or uncooked cabbage, Brussels sprouts, raw carrots, cooked carrots, mixed vegetables, yams or sweet potatoes, yellow or winter squash, eggplant or zucchini or other summer squash, kale or mustard or chard greens, cooked spinach, raw spinach, iceberg or head lettuce, romaine or leaf lettuce, green pepper, cucumber, celery, mushrooms, alfalfa sprouts, corn	Positive scores	Reverse scores
Nuts	Nuts, peanut butter	Positive scores	Reverse scores
Legumes	String beans, tofu or soybeans, beans or lentils, peas or lima beans	Positive scores	Reverse scores
Vegetable oil	Vegetable oil, oil-based salad dressing	Positive scores	Reverse scores

© 2024 Sotos-Prieto M et al. *JAMA Network Open*.

Tea and coffee	Tea, coffee, decaffeinated coffee or non-caffeinated tea	Positive scores	Reverse scores
Less healthy plant foods			
Refined grains	Refined grain breakfast cereal, white bread, English muffins or bagels or rolls, muffins or biscuits, white rice, pancakes or waffles, crackers, pasta	Reverse scores	Positive scores
Potatoes	French fries, baked or boiled or mashed potatoes, potato or corn chips	Reverse scores	Positive scores
Sugar-sweetened beverages	Soda with sugar, noncarbonated fruit drinks with sugar	Reverse scores	Positive scores
Fruit juices	Apple cider (nonalcoholic) or juice, orange juice, grapefruit juice, other fruit juice	Reverse scores	Positive scores
Sweets and desserts	Chocolates, candy bars, candy without chocolate, cookies (home-baked and ready-made), brownies, doughnuts, cake (home-made and ready-made), sweet roll or coffee cake or other pastry, pie (home-baked and ready-made), jams or jellies or preserves or syrup or honey	Reverse scores	Positive scores
Animal foods			
Animal fat	Butter added to food, butter or lard used for cooking	Reverse scores	Reverse scores
Dairy	Skim low fat milk, whole milk, cream, sour cream, sherbet, ice cream, yogurt, cottage or ricotta cheese, cream cheese, other cheese	Reverse scores	Reverse scores
Eggs	Eggs	Reverse scores	Reverse scores

© 2024 Sotos-Prieto M et al. *JAMA Network Open*.

Fish and seafood	Canned tuna, dark meat fish, other fish, shrimp or lobster or scallops	Reverse scores	Reverse scores
Meat	Chicken or turkey with skin, chicken or turkey without skin, bacon, hot dogs, processed meats, hamburger, liver, beef or pork or lamb as a mixed dish, beef or pork or lamb as a main dish	Reverse scores	Reverse scores
Miscellaneous animal-based foods	Pizza, chowder or cream soup, mayonnaise or other creamy salad dressing	Reverse scores	Reverse scores

Abbreviations: hPDI, healthful plant-based diet index, uPDI, unhealthful plant-based diet index; NHS, Nurses' Health Study.

© 2024 Sotos-Prieto M et al. *JAMA Network Open*.

eTable 2. Relative risks (95% confidence interval) for hip fracture according to quintiles of cumulative modified-healthful plant-based diet scores in the Nurses' Health Study (n= 70,285 postmenopausal)

	Quintile 1 (lowest)	Quintile 2	Quintile 3	Quintile 4	Quintile 5	P-trend value
**Healthful Plant-Based Diet Index with yogurt (positive score)**						
No. of cases/persons-years	350/277,395	420/310,302	424/312,633	421/320,734	423/317,038	
Multivariable model	Ref. 1.	1.03 (0.89, 1.19)	0.99 (0.86, 1.15)	0.94 (0.81, 1.09)	0.92 (0.78, 1.07)	0.19
**Healthful Plant-Based Diet Index with fish (positive score)**						
No. of cases/persons-years	347/280,332	394/304,102	450/318,641	420/319,319	427/315,707	
Multivariable model	Ref. 1.	1.01 (0.87, 1.17)	1.07 (0.92, 1.23)	0.98 (0.84, 1.14)	0.98 (0.84, 1.15)	0.66
**Healthful Plant-Based Diet Index with poultry (positive score)**						
No. of cases/persons-years	360/277,128	397/307,316	435/314,022	448/325,644	398/313,991	
Multivariable model	Ref. 1.	0.97 (0.83, 1.12)	1.00 (0.86, 1.16)	0.99 (0.85, 1.16)	0.89 (0.75, 1.05)	0.20
**Healthful Plant-Based Diet Index with fish, poultry, and yogurt (positive score)**						
No. of cases/persons-years	398/274,991	416/300,622	438/318,957	415/326,772	371/316,760	

© 2024 Sotos-Prieto M et al. *JAMA Network Open*.

Multivariable model	Ref. 1.	0.96 (0.83, 1.10)	0.98 (0.85, 1.13)	0.93 (0.80, 1.09)	0.86 (0.73, 1.02)	0.21

Multivariable model: age and energy intake, physical activity, BMI, height, history of diabetes, smoking, alcohol intake, post-menopausal hormone use, thiazides, furosemide-like diuretics, anti-inflammatory steroids, multivitamin supplements, supplemental intakes of calcium, vitamin D and retinol.

© 2024 Sotos-Prieto M et al. *JAMA Network Open*.

eTable 3. Relative risks (95% confidence interval) for hip fracture according to quintiles of cumulative food categories scores in the Nurses' Health Study (n= 70,285, postmenopausal)

	Quintile 1 (lowest)	Quintile 2	Quintile 3	Quintile 4	Quintile 5	P-trend value
**Total plant-food intake**						
Cases/persons-years	344/271,220	411/310,751	443/323,124	439/323,849	401/309,157	
Multivariable-adjusted model	Ref. 1.0	1.07 (0.92, 1.25)	1.13(0.95, 1.34)	1.07 (0.89, 1.29)	1.04 (0.85, 1.28)	0.72
**Healthful plant-food intake**						
Cases/persons-years	358/265,538	389/306,135	454/321,171	436/325,997	401/319,260	
Multivariable-adjusted model	Ref. 1.0	0.96 (0.82 1.11)	1.05 (0.90, 1.21)	1.00 (0.86, 1.16)	0.95 (0.80, 1.12)	0.74
**Less healthy plant food intake**						
Cases/persons-years	383/279,517	414/324,774	404/326,745	447/318,456	390/288,608	
Multivariable-adjusted model	Ref. 1.0	0.93 (0.80, 1.08)	0.89 (0.75, 1.05)	0.98 (0.82, 1.17)	0.97 (0.79, 1.19)	0.94
**Animal food intake**						
Cases/persons-years	434/286,254	453/315,848	420/323,164	378/320,887	353/291,947	
Multivariable-adjusted model	Ref. 1.0	1.01 (0.88, 1.15)	0.98 (0.84, 1.13)	0.92 (0.78, 1.08)	0.95 (0.80, 1.15)	0.45

Multivariable-adjusted model: age and energy intake physical activity, BMI, height, history of diabetes, smoking, alcohol intake, post-menopausal hormone use, thiazides, furosemide-like diuretics, anti-inflammatory steroids, multivitamin supplements, supplemental intakes of calcium, vitamin D and retinol.

© 2024 Sotos-Prieto M et al. *JAMA Network Open*.

eTable 4. Relative risks (95% confidence interval) for hip fracture according to quintiles of cumulative plant-based diet indices with different latency periods in the Nurses' Health Study*

	2y latency	6y latency	10y latency
**Healthful Plant-Based Diet**			
Quintile 1 (lowest)	Ref 1.0	Ref 1.0	Ref 1.0
Quintile 2	1.06 (0.91, 1.23)	1.14 (0.97, 1.35)	1.21 (1.00, 1.46)
Quintile 3	0.96 (0.82, 1.13)	1.08 (0.91, 1.28)	1.06 (0.88, 1.29)
Quintile 4	1.03 (0.88, 1.21)	1.08 (0.90, 1.28)	1.18 (0.97, 1.43)
Quintile 5	0.95 (0.81, 1.12)	1.00 (0.83, 1.20)	1.11 (0.91, 1.36)
P-trend	0.46	0.59	0.52
**Unhealthful Plant-Based Diet**			
Quintile 1 (lowest)	Ref 1.0	Ref 1.0	Ref 1.0
Quintile 2	1.04 (0.89, 1.21)	1.07 (0.91, 1.25)	1.03 (0.87, 1.23)
Quintile 3	1.03 (0.89, 1.20)	0.96 (0.81, 1.13)	0.97 (0.81, 1.16)
Quintile 4	1.10 (0.94, 1.28)	1.05 (0.89, 1.24)	0.90 (0.75, 1.08)
Quintile 5	1.05 (0.89, 1.23)	0.97 (0.81, 1.15)	0.97 (0.80, 1.18)
P-trend	0.43	0.68	0.37

Adjusted for: age and energy intake, physical activity, BMI, height, history of diabetes, physical activity, smoking, alcohol intake, post-menopausal hormone use, thiazides, furosemide-like diuretics, anti-inflammatory steroids, multivitamin supplements, supplemental intakes of calcium, vitamin D and retinol.

© 2024 Sotos-Prieto M et al. *JAMA Network Open*.

eTable 5. Relative Risks (95% confidence interval) for hip fracture according to quintiles of cumulative Plant-Based Diet Indices with different latency periods in the Nurses' Health Study*

	Quintile 1 (lowest)	Quintile 2	Quintile 3	Quintile 4	Quintile 5	P-trend value	P-for interaction
**Healthful Plant-Based Diet**							
**Age**							
**Age<75 years**							0.73
Multivariable-adjusted model	Ref. 1.	1.08 (0.88, 1.31)	0.99 (0.81, 1.22)	1.03 (0.84, 1.27)	0.86 (0.69, 1.07)	0.16	
**Age≥75 years**							
Multivariable-adjusted model	Ref. 1.	1.18 (0.96, 1.47)	0.96 (0.77, 1.21)	1.10 (0.88, 1.37)	1.11 (0.88, 1.40)	0.66	
**BMI (Kg/m^2)**							0.12
**BMI <25 Kg/m^2**							
Multivariable-adjusted model	Ref. 1.	1.15 (0.94, 1.40)	0.99 (0.81, 1.21)	0.99 (0.81, 1.23)	0.94 (0.76, 1.16)	0.20	
**BMI ≥25Kg/m^2**							
Multivariable-adjusted model	Ref. 1.	1.10 (0.88, 1.37)	0.96 (0.76, 1.21)	1.17 (0.93, 1.46)	1.06 (0.83, 1.36)	0.51	
**Physical Activity**							0.06

© 2024 Sotos-Prieto M et al. *JAMA Network Open*.

**Physical activity <median (9.3 MET-h/wk)**							
Multivariable-adjusted model	Ref. 1.	1.11 (0.91, 10.34)	0.99 (0.80, 1.21)	1.06 (0.86, 1.31)	1.01 (0.81, 1.27)	0.95	
**Physical activity≥median (9.3 MET-h/wk)**							
Multivariable-adjusted model	Ref. 1.	1.15 (0.91, 1.43)	0.97 (0.77, 1.22)	1.08 (0.86, 1.36)	0.98 (0.78, 1.23)	0.56	
**Unhealthful Plant-Based Diet**							
**Age**							0.13
**Age<75 years**							
Multivariable-adjusted model	Ref. 1.	0.86 (0.71, 1.06)	0.94 (0.77, 1.14)	1.00 (0.82, 1.23)	1.02 (0.82, 1.26)	0.46	
**Age≥75 years**							
Multivariable-adjusted model	Ref. 1.	1.18 (0.94, 1.47)	1.15 (0.92, 1.44)	1.19 (0.95, 1.49)	1.05 (0.82, 1.33)	0.83	
**BMI (Kg/m^2)**							
**BMI <25Kg/m^2**							0.08

© 2024 Sotos-Prieto M et al. *JAMA Network Open*.

Multivariable-adjusted model	Ref. 1.	1.10 (0.90, 1.34)	0.90 (0.73, 1.11)	1.10 (0.90, 1.34)	1.00 (0.81, 1.24)	0.98	
**BMI ≥25Kg/m^2**							
Multivariable-adjusted model	Ref. 1	0.89 (0.70, 1.11)	1.22 (0.98, 1.52)	1.07 (0.85, 1.34)	1.00 (0.78, 1.28)	0.51	
**Physical Activity**							0.73
**Physical activity <median (9.3 MET-h/wk)**							
Multivariable-adjusted model	Ref. 1	1.04 (0.82, 1.33)	1.16 (0.92, 1.46)	1.17 (0.93, 1.48)	1.05 (0.83, 1.33)	0.61	
**Physical activity≥median (9.3 MET-h/wk)**							
Multivariable-adjusted model	Ref. 1	1.00 (0.83, 1.21)	0.95 (1.77, 1.16)	1.04 (0.85, 1.28)	1.04 (0.83, 1.30)	0.66	

Multivariable-adjusted model: age and energy intake physical activity, BMI, height, history of diabetes, smoking, alcohol intake, post-menopausal hormone use, thiazides, furosemide-like diuretics, anti-inflammatory steroids, multivitamin supplements, supplemental intakes of calcium, vitamin D and retinol

© 2024 Sotos-Prieto M et al. *JAMA Network Open*.
