# Supplementary material for: Plant-Based Diets and Risk of Hip Fracture in Postmenopausal Women
Source: JAMA Netw Open. 2024 Feb 29;7(2):e241107. doi: 10.1001/jamanetworkopen.2024.1107 (PMC10905300; doi:10.1001/jamanetworkopen.2024.1107)
Supplement: Supplement 2. — Data Sharing Statement [file jamanetwopen-e241107-s002.pdf]

Data Sharing Statement

Sotos-Prieto. Plant-Based Diets and Risk of Hip Fracture in Postmenopausal Women. *JAMA Netw Open*. Published March 04, 2024. doi:10.1001/jamanetworkopen.2024.1107

Data
Data available: No
